# Lung aeration reduces blood pressure surges caused by umbilical cord milking in preterm lambs

**DOI:** 10.3389/fped.2023.1073904

**Published:** 2023-03-21

**Authors:** Douglas A. Blank, Kelly J. Crossley, Alison Thiel, Karyn A. Rodgers, Valerie Zahra, Martin Kluckow, Andrew W. Gill, Graeme R. Polglase, Stuart B. Hooper

**Affiliations:** ^1^ The Ritchie Centre, Hudson Institute of Medical Research, Melbourne, VIC, Australia; ^2^ Monash Newborn, Monash Children's Hospital, Melbourne, VIC, Australia; ^3^ The Department of Paediatrics, Monash University, Melbourne, VIC, Australia; ^4^ The Department of Obstetrics and Gynaecology, Monash University, Melbourne, VIC, Australia; ^5^ Department of Neonatology, Royal North Shore Hospital and University of Sydney, Sydney NSW, Australia; ^6^ Centre for Neonatal Research and Education, University of Western Australia, Perth, WA, Australia

**Keywords:** umbilical cord milking, delayed cord clamping, neonatal resuscitation, placental transfusion, prematurity, mechanical ventilalion

## Abstract

**Background:**

Umbilical cord milking (UCM) at birth causes surges in arterial blood pressure and blood flow to the brain, which may explain the high risk of intraventricular haemorrhage (IVH) in extremely preterm infants receiving UCM. This high risk of IVH has not been reported in older infants.

**Objective:**

We hypothesized that lung aeration before UCM, reduces the surge in blood pressure and blood flow induced by UCM.

**Methods:**

At 126 days' gestation, fetal lambs (*N* = 8) were exteriorised, intubated and instrumented to measure umbilical, pulmonary, cerebral blood flows, and arterial pressures. Prior to ventilation onset, the umbilical cord was briefly (2–3 s) occluded (8 times), which was followed by 8 consecutive UCMs when all physiological parameters had returned to baseline. Lambs were then ventilated. After diastolic pulmonary blood flow markedly increased in response to ventilation, the lambs received a further 8 consecutive UCMs. Ovine umbilical cord is shorter than the human umbilical cord, with ∼10 cm available for UCMs. Therefore, 8 UCMs/occlusions were done to match the volume reported in the human studies. Umbilical cord clamping occurred after the final milk.

**Results:**

Both umbilical cord occlusions and UCM caused significant increases in carotid arterial blood flow and pressure. However, the increases in systolic and mean arterial blood pressure (10 ± 3 mmHg vs. 3 ± 2 mmHg, *p* = 0.01 and 10 ± 4 mmHg vs. 6 ± 2 mmHg, *p* = 0.048, respectively) and carotid artery blood flow (17 ± 6 ml/min vs. 10 ± 6 ml/min, *p* = 0.02) were significantly greater when UCM occurred before ventilation onset compared with UCM after ventilation.

**Conclusions:**

UCM after ventilation onset significantly reduces the increases in carotid blood flow and blood pressure caused by UCM.

## Introduction

Deferred umbilical cord clamping (DCC) confers multiple benefits for preterm newborns, including lower risk of intraventricular hemorrhage (IVH) and improved hemodynamics (1–3). Until recently, these benefits were largely attributed to placental transfusion, due to an expected net movement of blood from the placenta into the infant after birth. As international guidelines recommend early cord clamping (ECC) for infants needing respiratory support at birth, it has been suggested that umbilical cord milking (UCM) can replicate the placental transfusion associated with DCC with no delay providing respiratory support (4, 5).

UCM involves milking a 20 cm length of umbilical cord over 2–3 s, 3–4 times (4). While clinical trials have suggested that UCM is safe, appears to confer the same benefits as DCC, and may be more effective in infants delivered by caesarean section, these trials mostly occurred in near term infants not requiring resuscitation (3–11). However, we have shown that UCM causes large surges in carotid arterial blood pressure and flow in preterm lambs, which potentially increases the risk of IVH (12). This is consistent with the recent finding of a 4-fold increase in severe IVH rates in extremely preterm infants receiving UCM, leading to the recommendation against UCM in these infants (13, 14). The question as to why it may be safe in some infants, but not in others, is currently unknown.

It is now clear that DCC has multiple benefits that are independent of placental transfusion (15). Physiologically-based cord clamping (PBCC) is deferring umbilical cord clamping (UCC) until after the lungs have aerated, pulmonary vascular resistance (PVR) has decreased and pulmonary blood flow (PBF) has increased (15). The rapid increase in arterial blood pressure (∼30%) and reduction in cardiac output (∼60%) caused by ECC are greatly reduced in preterm lambs managed with PBCC (2, 16). The increase in PBF takes over the role of providing venous return and left ventricular preload that is lost following umbilical cord clamping. In contrast, ECC prior to lung aeration causes a reduction in cardiac output and large increases in arterial blood pressure due to a sudden increase in systemic vascular resistance (SVR) and a loss of ventricular preload. Similarly, we have shown that UCM prior to lung aeration causes a large increase in arterial pressure during each milk, which returns to control pressures between milks, essentially creating repeated occlusions that simulate ECC with each milk (12).

In this study, we hypothesized that aerating the lung and increasing PBF prior to UCM, reduces the pressure surges that occur during UCM prior to lung aeration. We also hypothesise that repeating UCMs cause these changes by repeatedly occluding the umbilical cord blood flow, similar to repeating clamping and unclamping the umbilical cord. It is possible that the higher risk of IVH associated with UCM in extremely preterm infants is related to both the fragility of the germinal matrix and the inability to quickly aerate the lungs after birth (17). If correct, the increased risk of IVH associated with UCM may also remain high in older preterm infants whose lungs are not aerated at the time of UCM.

## Methods

All experimental procedures were performed in accordance with the National Health and Medical Research Council Code of Practice for the Care and Use of Animals for Scientific Purposes and were approved by the Monash University Animal Ethics Committee (MMCA/2019/09).

### Pre-experimental surgery and instrumentation

At 126 ± 1 days gestation (∼26 weeks in human fetus or neonate), anaesthesia was induced in pregnant ewes with 5% sodium thiopentone (i.v. Pentothal; 1 g in 20 ml) and maintained, following intubation, with inhaled isoflurane (1.5%–3%) in oxygen/air (2, 16). Preterm lambs were partially exteriorised (head and chest) by hysterotomy and polyvinyl catheters (20 gauge) were inserted into the left carotid artery and jugular vein. Flow probes (Transonic Systems; NY, United States) were placed around the right carotid artery, left main pulmonary artery, one umbilical artery and one (of two) umbilical veins. The trachea was intubated with a 4 mm cuffed endotracheal tube and lung liquid was passively drained prior to ventilation.

### Experimental approach

#### Umbilical cord occlusions and UCM prior to ventilation onset

The first sequence of umbilical cord interventions commenced following instrumentation and delivery of the lamb and after a period during which physiological parameters had stabilised. Prior to lung aeration, an investigator (DB) briefly occluded the umbilical cord for 2-3 s, 8 times, which did not involve UCM (Supplementary video). Following a brief recovery period (2–3 min), during which flows and pressures returned to control levels, UCM commenced. An investigator (DB) milked a 10 cm segment of umbilical cord 8 times, taking 2-3 s per milk, starting at the placental end and milking towards the lamb (Supplementary video). After each milk, the umbilical cord was released, which we have previously described as “UCM without placental refill.”(12) The umbilical cord was milked 8 times in our lambs because it is shorter than in humans (milked region 10 cm vs. 20 cm in humans) and so to achieve a similar volume of placental transfusion we increased the number of milks.

#### UCM after ventilation onset

Following the initial UCM, umbilical blood flows and arterial blood pressure were allowed to return to control levels before ventilation of the lamb commenced. Ventilation began with a sustained inflation (30 cmH_2_O x 30 s), followed by volume guaranteed mechanical ventilation at 7 ml/kg with a positive end-expiratory pressure of 5 cmH_2_O, rate of 60 inflations per minute, inspiratory time of 0.5 s, and fraction of inspired oxygen (FiO_2_) of 0.21 (Dräeger Babylog 8000 + ventilator, Dräeger, Lübeck, Germany). The peak inflation pressure (PIP) was limited to a maximum of 40 cmH_2_O. Lung aeration results in a rapid and sustained increase in PBF as previously described, resulting in forward flow into the lung during diastole; prior to ventilation onset this flow is mostly retrograde (away from the lungs) during diastole (18). When there was continuous PBF flow into the lung throughout the cardiac cycle (indicating left-to-right ductal shunting during diastole), UCM was repeated 8 times, exactly as occurred during UCM prior to mechanical ventilation. The umbilical cord was clamped and cut immediately after the final milk and an alfaxane (5–15 mg/kg/hr in 5% dextrose, Jurox, Rutherford, Australia) infusion commenced.

#### Data collection and monitoring

Blood gases were measured immediately prior to commencing the experiment and then at 10 minute intervals (ABL30, Radiometer, Copenhagen, Denmark). Ventilator and FiO_2_ adjustments were made as needed to maintain arterial pH >7.25, PaCO_2_ 45–55, and SpO_2_ according to published reference ranges for the 1st ten minutes, then 85%–95% thereafter (19). Vital signs and physiological parameters were monitored and recorded continuously using LabChart (ADInstruments, NSW, Australia).

All lambs were ventilated for 30 min. Ewes were euthanized following UCC whereas lambs were euthanized at the conclusion of the experiment, both with sodium pentobarbitone, (100 mg/kg IV, Jurox, Rutherford, Australia).

#### Analysis and statistics

Blood pressure (BP), mean PBF, mean carotid artery blood flow (CBF), and mean umbilical artery blood flow were measured from heartbeat to heartbeat. We measured blood flow, in ml/min, during the intervention in each vessel in relation to its baseline, before each occlusion and milking period. The net umbilical blood flow was to the fetal lamb was calculated during UCM by subtracting the umbilical artery flow from the umbilical venous flow ml/min.

Normal baseline and fetal data are presented as means and standard mean error (SEM) and non-normal data as medians and interquartile range. A one-way repeated measures analysis of variance (ANOVA) or a Friedman's test was used to compare baseline values prior to each intervention (occlusions, UCM pre-ventilation, and UCM post-ventilation) and the changes in blood pressure and blood flow during the intervention, as well as the maximum changes in blood pressure and blood flow during each occlusion or milk. A two-way repeated measures analysis of variance (ANOVA) with a post-hoc analysis using Bonferroni correction for multiple comparisons was used to compare continuous variables over time during the interventions, ie BP during occlusions or UCM before and after ventilation. IBM SPSS V27 was used for all statistical calculations. Statistical significance was accepted as *P* < 0.05.

## Results

### Baseline characteristics

Baseline values (measured before occlusions/UCM) for systolic BP were similar before and after ventilation onset, whereas baseline mean and diastolic BPs were lower after ventilation onset compared with before ventilation onset (*p* = 0.002 and *p* < 0.001, respectively; Table 1). In response to ventilation, baseline PBF significantly increased from 29 ± 13 ml/min before ventilation onset to 230 ± 23 ml/min (*p* < 0.001) immediately prior to UCM post-ventilation (822 ± 99 s after ventilation onset). Baseline net umbilical blood flows were similar between timepoints (*p* = 0.25). Baseline umbilical venous and arterial blood flows decreased significantly in response to ventilation onset and were lower prior to UCM post-ventilation than prior to occlusions and UCM before ventilation onset (*p* = 0.002 and *p* < 0.001, respectively). The partial pressure of carbon dioxide and lactate levels were similar prior to occlusions and UCM before ventilation onset compared to levels prior to UCM after ventilation onset. Partial pressure of oxygen increased significantly in response to ventilation (pre-ventilation = 24 ± 2 mmHg vs. post-ventilation = 40 ± 6 mmHg, *p* = 0.04) and there was a small increase in pH (pre-ventilation = 7.31 ± 0.02 vs. post-ventilation = 7.34 ± 0.01, *p* = 0.01). There was no difference in the mean time to complete the series of UCM before ventilation vs. after ventilation (41 ± 2 s vs. 45 ± 4 s, *p* = 0.3).

**Table 1:** Baseline characteristics and baseline measurements prior to umbilical cord intervention. UCM = umbilical cord milking.

Number of Subjects	8
Percent male	50%
Median Weight (Interquartile Range)	3.4 kg (3.3–3.6)
Gestational Age	125–127 days
Time between interventions, mean ± standard error of the mean	
– Occlusion pre-ventilation and UCM pre-ventilation	153 ± 39 s
– UCM pre-ventilation and starting mechanical ventilation	256 ± 47 s
– Starting mechanical ventilation and UCM post-ventilation	822 ± 99 s
Baseline values prior to UCM, mean ± standard error of the mean	
Baseline systolic blood pressure	
– Occlusion pre-ventilation	61 ± 3 mmHg
– UCM pre-ventilation	60 ± 3 mmHg
– UCM post-ventilation	58 ± 3 mmHg
Baseline mean blood pressure	
– Occlusion pre-ventilation	50 ± 2 mmHg
– UCM pre-ventilation	49 ± 3 mmHg
– UCM post-ventilation	**41 **±** 3** mmHg*
Baseline diastolic blood pressure	
– Occlusion pre-ventilation	40 ± 3 ml/min
– UCM pre-ventilation	41 ± 2 mmHg
– UCM post-ventilation	**29 **±** 2** mmHg*
Baseline carotid artery blood flow	
– Occlusion pre-ventilation	51 ± 3 ml/min
– UCM pre-ventilation	55 ± 3 ml/min
– UCM post-ventilation	**27 **± 2 ml/min*
Baseline pulmonary blood flow	
– Occlusion pre-ventilation	40 ± 12 ml/min
– UCM pre-ventilation	28 ± 12 ml/min
– UCM post-ventilation	**228 **±** 23 ml/min***^α^*
Baseline umbilical venous blood flow	
– Occlusion pre-ventilation	203 ± 31 ml/min
– UCM pre-ventilation	197 ± 21 ml/min
– UCM post-ventilation	135 ± 17 ml/min
Baseline umbilical arterial blood flow	
– Occlusion pre-ventilation	240 ± 26 ml/min
– UCM pre-ventilation	237 ± 24 ml/min
– UCM post-ventilation	**150 **± 14 ml/min*
Baseline net umbilical blood flow	
– Occlusion pre-ventilation	−37 ± 15 ml/min
– UCM pre-ventilation	−41 ± 21 ml/min
– UCM post-ventilation	−15 ± 23 ml/min

*, UCM post-ventilation is significantly lower than the values during brief occlusions or UCM pre-ventilation, *p* < 0.05, using Bonferroni adjustments for multiple comparisons.

^α^ , UCM post-ventilation is significantly higher than the values during brief occlusions or UCM pre-ventilation, *p* < 0.001 using Bonferroni adjustments for multiple comparisons.

### Effect of umbilical cord occlusion and umbilical cord milking

**Figure 1:**
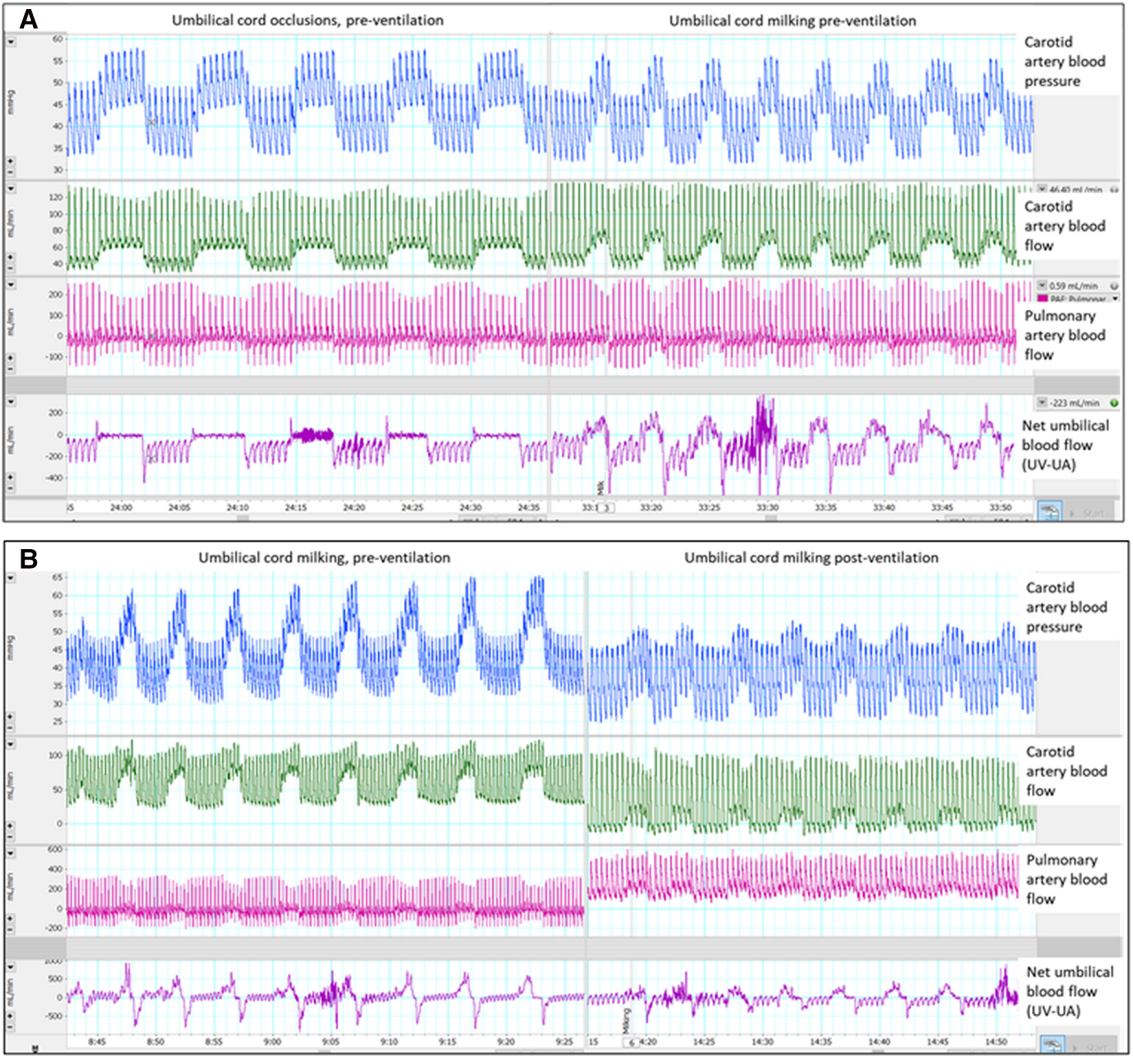
Single animal example of umbilical cord milking (UCM) and occlusions pre-ventilation and post-ventilation: Single animal example: (**A**) occlusions vs. umbilical cord milking pre-ventilation and (**B**) umbilical cord milking pre-ventilation vs. post-ventilation, UV = umbilical venous flow, UA = umbilical arterial flow.

**Figure 2:**
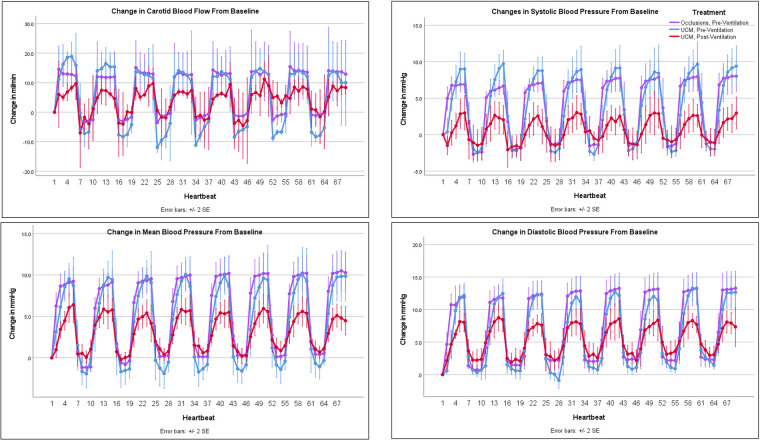
Changes in carotid blood flow and blood pressure with occlusions and umbilical cord milking (UCM): changes in carotid artery blood flow and blood pressure from baseline during umbilical cord occlusions pre-ventilation, UCM pre-ventilation, and UCM post-ventilation.

**Figure 3:**
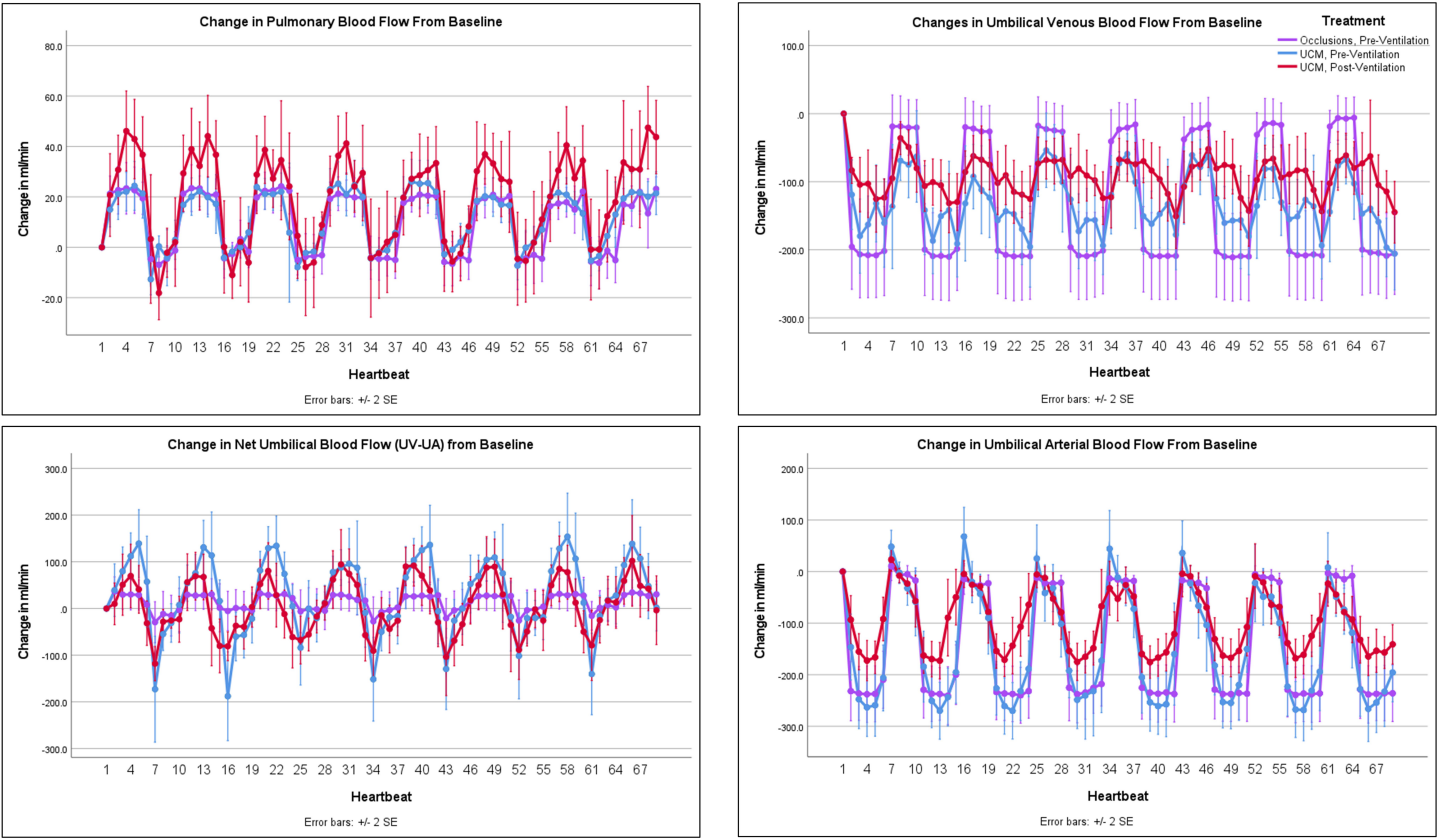
Change in pulmonary and umbilical blood flow with occlusions and umbilical cord milking (UCM): changes in pulmonary and umbilical blood flow from baseline during umbilical cord occlusions pre-ventilation, UCM pre-ventilation, and UCM post-ventilation.

Both before and after ventilation onset, each cord occlusion and each milk during UCM caused a significant increase in arterial BP (*p* < 0.001), CBF (*p* < 0.001) and PBF (*p* < 0.001), before they all decreased back to baseline values between occlusion/milk (Figures 1–3 and Supplementary video). Umbilical cord occlusions caused umbilical venous and arterial blood flows to cease (Figure 3 and Table 2). UCM also caused cessation of umbilical venous flow but caused retrograde flow in the umbilical artery both before (272 ± 81 ml/min, 115%) and after (177 ± 50 ml/min, 118%) ventilation, with flow quickly returning to baseline between milks.

**Table 2:** Average maximum changes in blood pressure (measured in the carotid artery) and blood flow with each umbilical cord occlusion or umbilical cord milk (UCM) and compared between sequences.

Average maximum change ± standard deviation, % change	Occlusions	UCM pre-ventilation	UCM post-ventilation
Blood pressure: average maximum increase with each occlusion or milk			
Systolic blood pressure	7.7 ± 2 mmHg, 13%	9.7 ± 3.2 mmHg, 17%	**3.3 ** **±** ** 2.4 mmHg** * **, 5%**
Mean blood pressure	10 ± 2.6 mmHg, 20%	10.3 ± 3.6 mmHg, 20%	**6.3 ** **±** ** 1.9 mmHg** * **, 15%**
Diastolic blood pressure	12.8 ± 3.2 mmHg, 33%	12.9 ± 3 mmHg, 32%	**9 ** **±** ** 2.5 mmHg** * **, 31%**
Carotid artery blood flow: average maximum increase with each occlusion or milk			
Carotid artery blood flow	17.2 ± 6.1 ml/min, 33%	16.7 ± 7.1 ml/min, 31%	**9.6 ** **±** ** 2.7 ml/min** ^α^ **, 37%**
Net umbilical blood flow: average increase with each occlusion or milk			
Net umbilical blood flow	16 ± 28 ml/min	25 ± 34.7 ml/min	1.6 ± 41.2 ml/min
Umbilical blood flow: average maximum decrease with each occlusion or milk			
Umbilical venous blood flow	211 ± 91 ml/min, 103%	199 ± 67 ml/min, 101%	149 ± 55 ml/min, 109%
Umbilical arterial blood flow	241 ± 77 ml/min, 100%	272 ± 81 ml/min, 115%	**177 **± 50 ml/min*, 118%

*, UCM post-ventilation is significantly lower than the values during brief occlusions or UCM pre-ventilation, *p* < 0.05 using Bonferroni adjustments for multiple comparisons.

^α^ , UCM post-ventilation is significantly lower than the values during brief occlusions, *p* < 0.05 using Bonferroni adjustments for multiple comparisons.

### Comparison of umbilical cord occlusions and UCM prior to ventilation onset

Umbilical cord occlusions and UCM prior to ventilation onset produced similar changes in BP, PBF, net umbilical blood flow, and umbilical venous and arterial blood flow (Figures 2 & 3 and Table 2). Pre-ventilation UCM increased the amplitude of the changes in CBF compared to occlusions (*p* = 0.01), due to lower flows between milks (Figure 2). In contrast the average maximum increase in CBF with each occlusion or milk was not different between interventions (17.2 ± 6.1 ml/min vs. 16.7 ± 7.1 ml/min, *p* = 1, Table 2), with both resulting in a 30% increase.

### UCM pre-ventilation vs. UCM post-ventilation

The lambs were ventilated for 822 ± 99 seconds before there was continuous PBF flow into the lung throughout the cardiac cycle and the sequence of UCM post-ventilation was performed. Before ventilation onset, UCM increased maximum systolic, mean, and diastolic BPs significantly more with each milk than UCM after ventilation onset (9.7 ± 3.2 mmHg vs. 3.3 ± 2.4 mmHg, *p* = 0.01, 10.3 ± 3.6 mmHg vs. 6.3 ± 1.9 mmHg, *p* = 0.048, and 12.9 ± 3 mmHg vs. 9 ± 2.5 mmHg, *p* = 0.045, respectively, Table 2). UCM after ventilation still produced significant fluctuations in the systolic, mean, and diastolic blood pressure compared to baseline values. Similarly, although the magnitude of the change in mean CBF caused by UCM was markedly greater prior to ventilation onset compared to after ventilation onset, the percentage changes were similar between UCM pre-ventilation and UCM post-ventilation (Table 2). This is simply because baseline CBFs post ventilation were ∼50% of baseline flows measured pre-ventilation (Table 1). UCM prior to ventilation onset caused a greater reduction in umbilical arterial blood flow, measured in ml/min, compared to UCM after ventilation (*p* = 0.003) onset because baseline arterial blood flows were significantly lower after ventilation onset (Figure 3, Table 2). Ventilation did not affect the changes in umbilical venous or net umbilical blood flow caused by UCM, with umbilical venous blood flows decreasing by ∼100% with UCM before and after ventilation.

## Discussion

Experimental studies have previously indicated that the large oscillations in carotid artery blood pressure and cerebral blood flow caused by UCM at birth could adversely affect newborn infants, by increasing the risk of IVH (12). A recent meta-analysis has subsequently found that UCM increases the risk of severe IVH compared with delayed cord clamping in preterm infants (14). As a result, the authors suggested that UCM should not be recommended for preterm infants <30 weeks gestational age. However, before UCM can be recommended for any infants at birth, we believe it is essential to understand why some infants, particularly very preterm infants, are highly susceptible to developing IVH in response to UCM, whereas others appear not so susceptible.

Our study confirms that UCM prior to lung aeration causes large and potentially dangerous fluctuations in arterial blood pressure and cerebral blood flow. We have also conclusively shown that aeration of the lung prior to UCM greatly reduces the amplitude of these oscillations in systolic, mean, and diastolic BPs and CBF. However, UCM, even after a prolonged period of ventilation, still causes concerning fluctuations in mean and diastolic BPs and CBF. Nevertheless, by mitigating the arterial pressure oscillations, it is possible that aeration of the lung prior to UCM reduces, but does not totally abolish, the risk of IVH in response to UCM.

Animal studies have shown that UCC prior to ventilation onset causes a large increase in arterial blood pressure, due to a sudden increase in SVR caused by occlusion of the umbilical arteries (2). As the placental circulation is a large, low resistance vascular bed, removing it from the newborn's systemic circulation substantially increases SVR. However, if the newborn has aerated its lungs, the subsequent decrease in PVR offers an alternative low resistance pathway for blood flow, which is evidenced by a large and rapid increase in left-to-right shunting across the ductus arteriosus after lung aeration (2). As a result, the pressure rise associated with cord clamping following lung aeration is greatly reduced and it is interesting to note that left-to-right shunting across the ductus arteriosus increased (indicated by increases in diastolic PBF) during each milk (Figure 1). This is caused by the brief increase in SVR during each milk which transiently increases the proportion of left ventricular output shunting left to right across the duct into the pulmonary circulation. As the increase in arterial pressure is the primary driver for the increase in CBF in response to UCC or UCM, reducing the amplitude of blood pressure fluctuations also reduces the amplitude of the fluctuations in CBF during UCM. Theoretically, this would reduce the risk of IVH (20).

The finding that brief occlusions of the umbilical cord produce identical increases in BP and CBF as UCM, demonstrates that these pressure and flow increases are due to umbilical artery occlusion rather than the act of milking blood toward the infant. The cessation of umbilical venous blood flow was similar during UCM and cord occlusions, whereas UCM caused a small retrograde movement of blood in the umbilical artery back towards the lamb. This results in a transient increase in net blood flow into the lamb as the umbilical arterial flow is blocked and the volume in the cord is pushed back towards the lamb. However, this net increase rapidly disappears as the umbilical arteries preferentially refills with blood from the pressurized lamb side rather than the low resistance placental side. This “refilling” of the umbilical arteries after each milk, likely explains the lower CBF between milks compared with flows measured between cord occlusions pre-ventilation (Figure 2). We have previously shown that the UCM technique of “milking without placental refill”, commonly used in clinical UCM trials, results in little to no net transfer of blood into the lamb as the cord refills mostly from the fetal side (12). The only UCM that produces an increase in blood volume would be the final milk because it is typically is followed by UCC. This likely explains why multiple milks with an intact cord produces similar transfusion volumes as a single milk utilising the cut-UCM technique (4, 21).

During PBCC, lung aeration and the resulting decrease in PVR caused umbilical artery and venous blood flows to decrease (12). At the same time PBF increased and blood flow across the ductus arteriosus became predominantly left-to-right, as indicated by antegrade PBF into the lungs during diastole (Figure 1) (18). The reduction in PVR redirects right ventricular output through the lungs, rather than shunting right-to-left across the ductus arteriosus, and also “steals” a portion of left ventricular output which shunts left-to-right across the ductus arteriosus resulting in elevated diastolic PBF (18). Thus, following lung aeration, the redirection of cardiac output through the lungs reduces umbilical artery blood flow which in turn reduces umbilical venous flow. However, there are no published clinical studies that have implemented a strategy of ensuring that the fetal to neonatal cardiopulmonary transition is so advanced before UCC, that flow across the ductus arteriosus is predominantly left to right prior to UCC. In very preterm infants even after the onset of effective spontaneous breathing, or adequate positive pressure ventilation, it is likely that PVR will be higher than occurred in our study, still resulting in fluctuations in BP and CBF. If the newborn is stable enough to achieve adequate lung aeration and pulmonary vascular relaxation prior to UCC, it is unlikely that UCM will have any benefit.

One limitation of this study is that all lambs were mechanically ventilated and so we did not examine the effects of spontaneous breathing on UCM pre- and post-ventilation. While most (∼90%) very preterm infants will initiate breathing prior to 1 min of age, it is unlikely that this breathing activity is sufficient to aerate their lungs and increase PBF before UCC (22). The ability of preterm newborns to aerate their lungs after birth varies based on factors like the severity of respiratory distress syndrome and respiratory drive. We chose to mechanically ventilate lambs because we wanted to avoid this variation. Our aim was to answer the scientific question, “does lung aeration prior to UCM reduce the large oscillations in blood pressure and carotid artery blood flow during UCM?” We compared the baseline values of blood pressure and blood flow prior to UCM/occlusions to blood pressure and blood flow during UCM/occlusions within each animal. The purpose of this study is to understand the physiological effects of UCM before and after lung aeration. Although these findings may have been strengthened by a larger cohort of lambs than we have studied, we are satisfied that our findings were clear and consistent between subjects. Reassuringly, our observations of carotid blood flow are consistent with the recently published experiment conducted by Chandrasekharan and colleagues investigating UCM before and after ventilation in asphyxiate preterm lambs (23).

## Conclusions

Our findings clearly demonstrate that lung aeration reduces the large oscillations in blood pressure and cerebral blood flow caused by UCM. Thus, as extremely preterm infants are unlikely to aerate their lungs prior to UCM, this explains why these infants are at higher risk of IVH following UCM. If correct, then the warning against UCM in infants less than 28 weeks gestation should be extended to all infants requiring resuscitation, as they are also unlikely to have had an opportunity to aerate their lungs prior to UCM.

## Data Availability

The raw data supporting the conclusions of this article will be made available by the authors, without undue reservation.
